# Factors associated with failure of using high flow nasal cannula in children

**DOI:** 10.1111/crj.13533

**Published:** 2022-08-29

**Authors:** Kanokkarn Sunkonkit, Supakanya Kungsuwan, Sukanlaya Seetaboot, Sanit Reungrongrat

**Affiliations:** ^1^ Division of Pulmonary and Critical Care, Department of Pediatrics, Faculty of Medicine Chiang Mai University Chiang Mai Thailand

**Keywords:** children, high flow nasal cannula, non‐invasive ventilation, respiratory support

## Abstract

**Introduction:**

High flow nasal cannula (HFNC) has significantly success in treating acute respiratory distress while HFNC failure dramatically increases mortality and morbidity.

**Objective:**

To describe factors associated with failure of HFNC use in children.

**Methods:**

We performed a retrospective observational study using demographic and laboratory findings. We compared clinical and laboratory variables in both successful and failed HFNC groups. The correlations between factors and HFNC failure were constructed by binary logistic regression analysis.

**Results:**

Between August 2016 and May 2018, 250 children receiving HFNC (median age 16 months; range 1–228 months, male 50.8%) were enrolled. Pneumonia was the most common cause of respiratory distress, and the median length of stay (LOS) in hospital was 11 days. HFNC failure was found 16.4% while HFNC complication was 4.8% including epistaxis, pressure sore, and gastric distension. Based on multivariable logistic regression analysis, factors associated with HFNC failure were children with congenital heart disease comorbidity (*p* = 0.005), HFNC use with maximum FiO_2_ > 0.6 (*p* = 0.021), lobar infiltration on chest X‐ray (*p* = 0.012), the reduction of heart rate, and respiratory rate <20% after 1 h of HFNC use (*p* = 0.001 and *p* = 0.001, respectively).

**Conclusion:**

HFNC is feasible to use for children with respiratory distress; however, patients with congenital heart disease, using HFNC with FiO_2_ > 0.6, lobar infiltration on chest X‐ray should be closely monitored. Heart rate and respiratory rate are important parameters in addition to clinical assessment for evaluating HFNC failure in children.

## Graphical abstract

HFNC seems to be well tolerated in children with respiratory distress; however, children with congenital heart disease, using HFNC with FiO_2_ > 0.6, having lobar infiltration on chest X‐ray and unimproved vital signs after HFNC use are factors associated with failure of using HFNC in children.

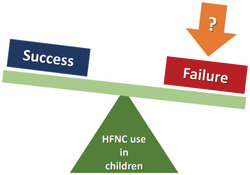

**Abbreviations**
BiPAP: bi‐level positive airway pressure
BW: body weight
CAP: community‐acquired pneumonia
CBC: complete blood count
CPAP: continuous positive airway pressure
FiO_2_
: fraction of inspired oxygen
GA: gestational age
HFNC: high flow nasal cannula
IQR: interquartile range
kg: kilograms
LOS: length of stay
LPM: liters per minute
NIV: non‐invasive ventilation
OR: odds ratio
pCO_2_
: partial pressure of carbon dioxide
PICU: pediatric intensive care unit
PRISM III: Pediatric Risk of Modified III scores
RR: relative risk
SLE: systemic lupus erythematosus
SpO_2_
: oxygen saturation

## 1 INTRODUCTION

High flow nasal cannula (HFNC) is extensively used as an important non‐invasive respiratory support.^1^, ^2^, ^3^, ^4^ Many studies show that most patients are more comfortable with HFNC than a face mask.^1^, ^2^, ^3^, ^5^ A high flow system, depending on the size and type of nasal cannula, refers to flow rate ≥2 L/min (LPM) despite a range from 4 to 70 LPM.^5^, ^6^ HFNC is composed of a heated and humidified mixture of oxygen and airflow, which is greater than the inspiratory flow of the patient, so the patient can obtain fixed fraction of inspired oxygen (FiO_2_).^6^, ^7^ In regard to respiratory support, HFNC has reported plenty of advantages to reduce the intubation and improve both oxygenation and ventilatory status.^1^, ^2^, ^3^, ^4^, ^5^, ^6^, ^8^ Previous studies have mentioned the mechanisms of HFNC including decreased inspiratory resistance, washout rebreathing of carbon dioxide gas, decreased metabolic work, maintaining positive airway pressure, and improving mucociliary clearance.^1^, ^4^, ^6^, ^8^, ^9^, ^10^, ^11^, ^12^, ^13^ Consequently, HFNC can improve oxygenation, reduce the respiratory rate, work of breathing, and endotracheal intubation rate.^14^, ^15^, ^16^ On the other hand, some adverse effects of HFNC are air leak syndrome (pneumothorax and pneumomediastinum), nasal bleeding, noise emissions, abdominal distension, and delayed intubation.^1^, ^17^, ^18^

Recent studies have demonstrated that the HFNC failure rate ranges from 6.0 to 24.4%^18^, ^19^, ^20^, ^21^, ^22^, ^23^, ^24^; however, a few studies demonstrated the risk factors of HFNC failure in children. This study aims to describe the factors associated with HFNC failure in children and complications in order to ensure and improve the management and outcome in the future.

### 1.1 Objectives

The first objective of our study was identifying factors associated with HFNC failure in children. The second objective was to characterize the complications from HFNC use.

## 2 MATERIALS AND METHODS

### 2.1 Study design

This was a single‐center, retrospective chart review of children using HFNC in a tertiary care hospital in pediatric intensive care (PICU) and outside PICU between August 2016 and May 2018 at Pediatric Department, Faculty of Medicine, Chiang Mai University hospital, Chiang Mai, Thailand. A retrospective review was approved by the Institutional Review Board at the Research Ethics Committee, Faculty of Medicine, Chiang Mai University hospital (Study code: PED‐2561‐05570), with a waiver of informed consent granted.

### 2.2 Patient population

Inclusion criteria was pediatric patients aged 1 month to 15 years of age who used HFNC at Chiang Mai University Hospital. Exclusion criteria was the pediatric patient who were on tracheostomy. The patient data including age, sex, date of admission and discharge from hospital, history of prematurity (defined as born prior to 37 weeks gestational age [GA]), history of intubation, underlying diseases, diagnosis, length of stay (LOS) in hospital, place where HFNC used, infiltration pattern on chest X‐ray, initial vital signs, and vital signs after HFNC use were collected. HFNC data including maximum total flow, FiO_2_, duration, outcome, and complication of HFNC use were recorded. HFNCs are available in both pediatric general ward and PICU setting. HFNC initiation and monitoring in this study were based on Figure S1. HFNC failure was defined as need for intubation or non‐invasive ventilation (NIV) support; however, continuous positive airway pressure (CPAP) and bi‐level positive airway pressure (Bi‐level PAP) were not available in our pediatric ward setting. Therefore, the pediatric patients who were prescribed HFNC and failed in our institute will be intubated and transfer to PICU.

### 2.3 High flow nasal cannula devices

HFNC therapy in our institution uses the GGM HF‐2900 Humidoflo® HFT system (Great Group Medical, Changhua, Taiwan). Enrollment for HFNC management was based on the decision of physicians following HFNC recommendations. The HFNC guidelines follow the Pediatric HFNC guideline at the Pediatric Pulmonary and Critical Care Division, Chiang Mai University Hospital (Figure S1). Regarding the guidelines, indications for using HFNC included hypoxemia with respiratory distress due to acute bronchiolitis or pneumonia, chronic lung disease, congestive heart failure, and respiratory support post‐extubation. The guidelines also suggested that contraindications included pneumothorax, congenital lung cyst, severe upper airway obstruction, and no spontaneous breathing. The cannula size was chosen, which did not occlude more than 50% of the internal diameter of each the nares. According to HFNC setting, the flow rate started at 2 L/min/kg and increased by 0.5 L/min/kg as work of breathing required or fair air entry, but not exceed total flow of 2 L/min/kg; FiO_2_ started at 0.6 and titrated as needed to achieve target oxygen saturation (SpO_2_); and set temperature at 34–37°C and was adjusted to the patient performance. After using HFNC, vital signs (heart rate, respiratory rate, and oxygen saturation [SpO_2_]), clinical status, work of breathing, chest X‐ray, and blood gas were monitored. HFNC use was discontinued when patients needed more respiratory support such as intubation or NIV. In the other hand, if the patients improved from their respiratory distress, HFNC weaning protocol would be considered.

### 2.4 Statistical analysis

Data analysis was carried out using IBM SPSS 23.0. The demographic data were evaluated by descriptive statistics. Normally distributed continuous variables were compared using Student's *t* tests, and skewed continuous variables were compared using Mann–Whitney *U* test. Categorical data were evaluated by chi‐square test or Fisher's exact test. A *p* value less than 0.05 indicated statistical significance. The correlations between factors and HFNC failure were constructed by logistic regression analysis.

## 3 RESULTS

### 3.1 Demographic data

Two hundred and fifty pediatric patients using HFNC due to respiratory distress were admitted between August 1, 2016, and May 31, 2018. Table 1 demonstrated the information regarding demographic and clinical data between HFNC success and the failure groups. According to the information, the median age was 16 months (interquartile rage [IQR]: 6–38.25), and half of the pediatric patients (127 patients; 50.5%) were males. Sixty‐one of 250 patients (24.4%) were born prior to GA 37 weeks while 17 patients (6.8%) had a history of intubation. The most common cause of respiratory distress was pneumonia found in 143 patients (57.2%). Other causes of respiratory distress were acute bronchiolitis in 53 patients (21.2%), post‐extubation stridor in 44 patients (17.6%), and congestive heart failure in 10 patients (4%). Half of the patients (50%) had underlying diseases. For the underlying diseases, congenital heart disease were identified in 73 patients (29.2%), chronic lung disease occurred in 21 patients (8.4%), neuromuscular disorder occurred in 12 patients (4.8%), systemic lupus erythematosus (SLE) occurred in 6 patients (2.4%), thalassemia occurred in 5 patients (2.0%), hematologic malignancy occurred in 5 patients (2.0%), and short bowel syndrome occurred in 3 patients (1.2%). Overall, using HFNC at general pediatric ward was equivalent to 52.4% (*n* = 131), while the failure of HFNC use following with intubation was 16.4% (*n* = 41). The median of intubation time was 26 h (IQR: 0–144). The median of maximum total flow rate and FiO_2_ used in both successful and failed groups were 1 L/min/kg (IQR: 1–2), 1.8 L/min/kg (IQR:1–2), FiO_2_ 0.6 (IQR:0.6–0.6), and 0.6 (IQR:0.6–0.7), respectively. The median LOS in hospital was 11 days (IQR; 7.0–23.25) while the median duration of HFNC use was 48 h (IQR; 19.0–72.0). On chest X‐ray, we found perihilar infiltration in 139 patients (55.6%), interstitial infiltration in 44 patients (17.6%), hyperinflation with atelectasis in 43 patients (17.2%), and lobar infiltration in 24 patients (9.6%). Most patients used HFNC without complication (95.2%); however, the complications from HFNC such as gastric distension in 7 patients (2.8%), nose pressure sore in 4 patients (1.6%), and epistaxis in 1 patient (0.4%) occurred.

**TABLE 1:** Baseline demographic data (*n* = 250)

Parameters	HFNC outcome		*p* value
	Success (*n* = 209)	Failure (*n* = 41)	
Age <2 years old, *n* (%)	117 (56.0)	30 (73.2)	*0.041* *
Sex (Male: Female)^a^	0.95: 1	1.56: 1	0.154
Body weight (kg)	8 (5.0, 12.0)^a^	7 (4.0, 10.0)^a^	0.075
History of preterm (GA < 37 weeks), *n* (%)	43 (20.6)	18 (43.9)	*0.001* *
History of intubation, *n* (%)	12 (5.7)	5 (12.2)	0.168
Diagnosis			*0.006* *
Pneumonia, *n* (%)	115 (55.0)	28 (68.3)	
Acute bronchiolitis, *n* (%)	49 (23.4)	4 (9.8)	
Post‐extubation stridor, *n* (%)	40 (19.1)	4 (9.8)	
Congestive heart failure, *n* (%)	5 (2.4)	5 (12.2)	
Underlying disease			*<0.001* *
No underlying disease, *n* (%)	125 (59.8)	0 (0.0)	
Chronic lung disease, *n* (%)	19 (9.1)	2 (4.9)	
Congenital heart disease, *n* (%)	44 (21.1)	29 (70.7)	
Others, *n* (%)	21 (10.0)	10 (24.4)	
HFNC use			
at ward, *n* (%)	116 (55.5)	15 (36.6)	*0.027* *
in PICU, *n* (%)	93 (44.5)	26 (63.4)	
Initial heart rate (/min)	145 (130.0, 160.0)^a^	150 (140.0, 155.0)^a^	0.514
Initial respiratory rate (/min)	48 (40.0, 52.0)^a^	48 (40.0, 54.0)^a^	0.629
Max. total flow rate (L/min/kg)	1 (1.0, 2.0)^a^	1.8 (1.0, 2.0)^a^	0.053
Max. FiO_2_	60 (60.0, 60.0)^a^	60 (60.0, 70.0)^a^	*< 0.001* *
Duration of HFNC use (hour)	48 (28.0, 84.0)^a^	4 (2.5, 9.0)^a^	*< 0.001* *
Hemoglobin (g/dl)	11 (9.8, 12.6)^a^	10.7 (9.2, 11.2)^a^	0.064
White blood cell count (cells/mm^3^)	10 160 (7780, 14 395)^a^	12 100 (8635, 16 205)^a^	0.156
Platelet count (cells/mm^3^)	253 000 (212 000, 316 000)^a^	267 000 (195 000, 312 000)^a^	0.550
Sodium (mmol/L)	136 (133.0, 138.0)^a^	135 (132.5, 138.0)^a^	0.255
Potassium (mmol/L)	3.8 (3.5, 4.2)^a^	3.7 (3.2, 4.2)^a^	0.109
Chloride (mmol/L)	100 (97.0, 103.0)^a^	98 (96.0, 103.0)^a^	0.471
Total CO_2_ (mmol/L)	20 (18.0, 25.0)^a^	20 (18.0, 27.0)^a^	0.761
Chest X‐ray			
Lobar infiltration, *n* (%)	8 (3.8)	16 (39.0)	*<0.001* *
Others, *n* (%)	201 (96.2)	25 (61.0)	
LOS in hospital (days)	8 (6.0, 21.0)^a^	22 (16.0, 41.5)^a^	*<0.001* *
Complication			
No complications, *n* (%)	199 (95.2)	39 (95.1)	0.933
Epistaxis, *n* (%)	1 (0.5)	0 (0.0)	
Pressure sore, *n* (%)	3 (1.4)	1 (2.4)	
Gastric distension, *n* (%)	6 (2.9)	1 (2.4)	

Abbreviations: dl, deciliter; FiO_2_, fraction of inspired oxygen; g, grams; GA, gestational age; HFNC, high flow nasal cannula; kg, kilograms, L, liter; LPM, liter per minute; LOS, length of stay; mm^3^, cubic millimeter.

^a^ Median (interquartile range; IQR).

* Statistically significant *p* < 0.05.

The comparison of baseline characteristic of patients between HFNC success and failure groups was shown in Table 1. The patients using HFNC successfully had older age (117 months [56%] vs. 30 [73.2%], *p* = 0.041) and history of preterm (43 patients [20.6%] vs. 18 patients [43.9%], *p* = 0.001) compared with the other group. More than half of both groups had a diagnosis of pneumonia. The second most common diagnosis in the HFNC success group was acute bronchiolitis while the other group had congestive heart failure. Most patients in the HFNC group had no underlying disease while nearly one‐third of the comorbidities of patients in the HFNC failure group had congenital heart disease comorbidity. Both patients in HFNC success and failure groups used HFNC at pediatric ward (116 patients [55.5%] vs. 15 patients [36.6%], *p* = 0.027). Moreover, baseline characteristics including maximum FiO_2_, duration of HFNC use, lobar infiltration on chest X‐ray, and LOS in hospital were different between the HFNC success and failure group with statistical significance. However, our study found that there were no statistical differences between groups in terms of sex, body weight (BW), history of intubation, initial heart rate, initial respiratory rate, maximum total flow rate, complete blood count (CBC), electrolyte, and complications.

### 3.2 Clinical parameters associated with HFNC failure

From the multivariable logistic regression analysis, patients being younger than 2 years of age, history of preterm birth, and using HFNC in PICU were not associated with HFNC failure. Among all the results, the presence of congenital heart comorbidity (RR 6.36, 95% CI, 1.74–23.17; *p* = 0.005), using HFNC with maximum FiO_2_ > 0.6 (RR 4.23, 95% CI, 1.24–14.37; *p* = 0.021), presence of lobar infiltration on chest X‐ray (RR 5.85, 95% CI, 1.48–23.18; *p* = 0.012), and reduction of heart rate and respiratory rate less than 20% after HFNC use for 1 h (RR 16.69, 95% CI, 3.39–82.27; *p* = 0.001 and RR 8.79, 95% CI, 2.47–31.26; *p* = 0.001, respectively) were dramatically associated with HFNC failure (Table 2).

**TABLE 2:** Factors associated with HFNC failure in children (*n* = 250)

Parameters	HFNC outcome		Relative risk (95% CI)	*p* value
	Success (*n* = 209)	Failure (*n* = 41)		
Age <2 years old	117 (56.0)	30 (73.2)	‐	0.950
History of preterm	43 (20.6)	18 (43.9)	‐	0.809
Congenital heart disease comorbidity	44 (21.1)	29 (70.7)	6.36 (1.74–23.17)	*0.005* *
HFNC use in PICU	93 (44.5)	26 (63.4)	‐	0.284
Max FiO_2_ > 0.6	32 (15.3)	14 (34.1)	4.23 (1.24–14.37)	*0.021* *
Decrease heart rate <20% after HFNC use for 1 h	4 (1.9)	22 (53.7)	16.69 (3.39–82.27)	*0.001* *
Decrease respiratory rate <20% after HFNC use for 1 h	10 (4.8)	28 (68.3)	8.79 (2.47–31.26)	*0.001* *
Lobar infiltration on chest X‐ray	8 (3.8)	16 (39.0)	5.85 (1.48–23.18)	*0.012* *

Abbreviations: FiO_2_, fraction of inspired oxygen; HFNC, high flow nasal cannula; PICU, pediatric intensive care unit.

* Statistically significant *p* < 0.05.

## 4 DISCUSSION

This study demonstrates that HFNC is an effective non‐invasive respiratory support in children. Overall, our study provides the information that HFNC use is favorable for respiratory distress in children starting age from 1 to 228 months, especially pneumonia and acute bronchiolitis without outstanding complications such as pneumothorax or pneumomediastinum. Our study found that patients without underlying disease had a greater HFNC success rate. We found that HFNC failure rate was 16.4%, compared with other studies ranging from 6 to 24.4%.^18^, ^19^, ^20^, ^21^, ^22^, ^23^, ^24^ Our study had two times higher HFNC failure rate than the data by Better at al.^21^ who studied 231 pediatric patients using HFNC outside PICU, retrospectively (HFNC failure rate was 6%). Vareesunthorn et al.^23^ described in a 3‐year retrospective study of 99 pediatric patients aged <5 years, admitted to a pediatric ward with the diagnosis of community‐acquired pneumonia (CAP) using modified HFNC with a failure rate of 7%. In contrast, our HFNC failure rate was less than Kallappa et al.^24^ who studied HFNC use in bronchiolitis patients for 3 years and found that intubation rate was 24.4%, approximately 1.5 times higher than our study. On the other hand, Bressan et al.^25^ who demonstrated in a prospective observational pilot study in 27 infants with acute bronchiolitis treated with HFNC in a general ward showed no negative outcome from HFNC. This study excluded infants with cardiac history, recurrent wheezing, chronic lung disease, neuromuscular disease, home oxygen dependence, and history of a tracheostomy, but our study included all of patients treated with HFNC with several underlying diseases, which caused our study to have a higher failed HFNC rate.^25^ Furthermore, this difference is due to local factors from physician decision, progression of disease, underlying diseases, and availability of resources such as nurse to patient ratio.

Based on the multivariable logistic regression analysis, we found that the presence of congenital heart comorbidity (*p* = 0.005), HFNC use with maximum FiO_2_ > 0.6 (*p* = 0.021), lobar infiltration on chest X‐ray (*p* = 0.012), and the reduction of heart rate and respiratory rate less than 20% after 1 h HFNC use (*p* = 0.001 and *p* = 0.001, respectively) were associated with increasing HFNC failure. However, our finding found no differences of HFNC failure rate in younger children than 2 years of age, preterm, using HFNC at ward or PICU, initial heart rate, and respiratory rate. Comparing with other studies, Better et al. retrospectively examined patients using HFNC outside PICU concluding that higher FiO_2_ requirement (OR = 38.3, 95% CI: 4.0–366.3; *p* = 0.002), diagnosis less likely to be bronchiolitis (OR = 0.3, 95% CI: 0.1–0.9; *p* = 0.048), history of intubation (*p* = 0.046), and cardiac comorbidities (*p* = 0.026) are related with HFNC failure.^21^ Vareesunthorn et al. with a 3‐year retrospective study in children <5 years implied that the SpO_2_/FiO_2_ ratio <264 and higher FiO_2_ requirement are associative predictors of HFNC failure.^23^ Abboud et al. studied 113 patients with viral bronchiolitis using HFNC in PICU found HFNC failure 18.6%, which was associated with initial respiratory rate (*p* < 0.001), pCO_2_ before and after HFNC use (*p* < 0.001) and (*p* < 0.001), respectively, and higher Pediatric Risk of Modified III scores (PRISM III) (*p* < 0.001).^22^ Based on our study, younger age and history of preterm were not associated with HFNC failure, which was similar to Betters^21^ and Abboud et al.^22^ studies.

For the HFNC setting, we found a maximum FiO_2_ impacted on the HFNC failure, especially more than 0.6 while the maximum total flow rate per kilogram was not associated with the failure and was similar to Betters^21^ and Vareesunthorn et al.^23^ studies. Betters et al. found that maximum flow (ml/kg) of HFNC was not different between HFNC success and the failure group (median 714.3 [IQR: 487.8–1041.7] vs. 629.1 [IQR: 476.2–1041.7], *p* = 0.787),^21^ and Vareesunthorn et al. revealed that maximum total flow rate per kilogram was not different between the success and failure groups (1.3 ± 0.4 vs. 1.5 ± 0.4, *p =* 0.358).^23^ Optimal total flow rate and FiO_2_ adjusted following clinical signs and symptoms bring about effective respiratory support. Higher FiO_2_ requirement is another factor associated with HFNC failure, which were found in Betters^21^ and Vareesunthorn et al.^23^ studies as well as our study. Moreover, those studies appeared that a high maximum FiO_2_ could predict HFNC failure (FiO_2_ 0.4 [IQR: 0.3–0.6] vs. FiO_2_ 1.0 [IQR: 0.6–1.0], *p* < 0.001 and FiO_2_ 0.38 ± 0.08 vs. FiO_2_ 0.53 ± 0.11, *p* < 0.001, respectively). Patients having more severity of disease or progression of disease require higher oxygen requirement or higher respiratory support such as invasive ventilation.^26^, ^27^ Our study revealed that the total flow rate was not related to HFNC failure; thus, FiO_2_ might be a substantial predictor for HFNC failure.

Children with congenital heart disease comorbidity and lobar infiltration on chest X‐ray both revealed significant values in the logistical regression analysis, and may demonstrate significance in the setting of more patients. Children with congenital heart disease may present with increased work of breathing and hypoxia which might be concealed by positive airway pressure and high FiO_2_ by HFNC.^21^ Thus, this patient group required closed monitoring and frequent reassessment after HFNC initiation.

Although initial heart rate and respiratory rate were not associated with HFNC failure in our study, the reduction of heart rate and respiratory rate less than 20% after 1 h HFNC use were associated with increasing HFNC failure significantly. This emphasizes that vital signs including heart rate and respiratory rate are important parameters in addition to clinical assessment for evaluating HFNC failure in children especially in the first hour after HFNC initiation.

This study shows the LOS in hospital was 11 days, however, with multifactorial issues impacting the duration, namely, clinical signs and symptoms of patients, progression of disease, cause of respiratory distress, other comorbidities, and different physician management. We illustrated that the failure group had less duration of HFNC use than the success group with statistical significance, which was similar to Betters et al. study (*p* = 0.015)^21^ because of early further intervention. The complications from HFNC in our study, unrelated to HFNC failure, such as gastric distension, nose pressure sore, and epistaxis, were comparable with Slain et al.^8^

Our current study suggested that HFNC is still an effective treatment in respiratory distress in children in order to improve the work of breathing and decrease intubation rates. HFNC can ameliorate the work of breathing with the mechanisms of HFNC such as inspiratory resistance reduction, washout rebreathing gas, decreased metabolic work, providing positive airway pressure, and improving mucociliary clearance.^1^, ^4^, ^7^, ^8^, ^9^, ^10^, ^11^, ^12^, ^13^ In addition, HFNC is easy to use, comfortable, and tolerable, which makes HFNC is attractive and broadly used in children and adults. However, we suggested that close monitoring should be considered in HFNC use, especially in high‐risk groups for early detection for non‐responders and not to delay intubation. The failure of HFNC and delaying intubation results in higher morbidity, mortality, and prolonged mechanical ventilation.^18^, ^28^

There are some notable limitations to our study that require consideration. First, this is a retrospective study, so some laboratory data such as electrolytes and some management were not available for analysis. Second, time of initiation, initial flow, and discontinuing HFNC are highly changeable based on clinical status of patients and dependent on physician experience, pleasure, and preference. Third, NIV was not available in our pediatric ward setting. The patients who were prescribed HFNC and failed will be intubated and transfer to PICU. So, the data regarding comparison of effectiveness and duration of CPAP and bi‐level PAP were not available. Finally, co‐treatment such as sedation techniques, feeding during HFNC, and IV fluid therapy, which may impact on decision making and HFNC failure, is unable to address in this current study design. Therefore, future prospective studies are required to illustrate the outcome of the HFNC guidelines, effects of co‐treatment, cost‐effectiveness, safety concerns, long‐term benefits, and socio‐economic impact in pediatric patients.

## 5 CONCLUSIONS

HFNC, a useful non‐invasive respiratory treatment, is practicable in children with respiratory distress. However, patient who have congenital heart disease comorbidity, using HFNC with FiO_2_ > 0.6, lobar infiltration on chest X‐ray, heart rate, and respiratory rate decreased less than 20% after HFNC use for 1 h should be closely monitored because of higher risk of HFNC failure. Heart rate and respiratory rate are important parameters in addition to clinical assessment for evaluating HFNC failure in children.

## AUTHOR CONTRIBUTIONS

Kanokkarn Sunkonkit, M.D., was responsible for the study concept, study design, interpretation of results, data collection, preparation of the first draft of the manuscript, data analysis, editing of manuscript drafts, and approval of the final version of the manuscript. Supakanya Kungsuwan, M.D., was responsible for the editing of manuscript drafts and approval of the final version of the manuscript. Sukanlaya Seetaboot, M.N.S., was responsible for the data collection and approval of the final version of the manuscript. Sanit Reungrongrat, M.D., was responsible for the editing of manuscript drafts and approval of the final version of the manuscript.

## CONFLICT OF INTEREST

The authors declare no conflict of interest.

## ETHICS STATEMENT

This study was approved by the Ethics Committee of the Faculty of Medicine, Chiang Mai University, Thailand (study code: PED‐2561‐05570). The requirement of informed consents was waived by the Ethics Committee of the Faculty of Medicine, Chiang Mai University, Thailand.

## Supporting information


**Figure S1:** HFNC initiation and monitoring in Pediatric Department, Chiang Mai University hospital

## Data Availability

Data are available on request due to privacy/ethical restrictions.
