# Supplementary figures and images for: Factors associated with failure of using high flow nasal cannula in children
Source: Clin Respir J. 2022 Aug 29;16(11):732–9. doi: 10.1111/crj.13533 (PMC9629993; doi:10.1111/crj.13533)

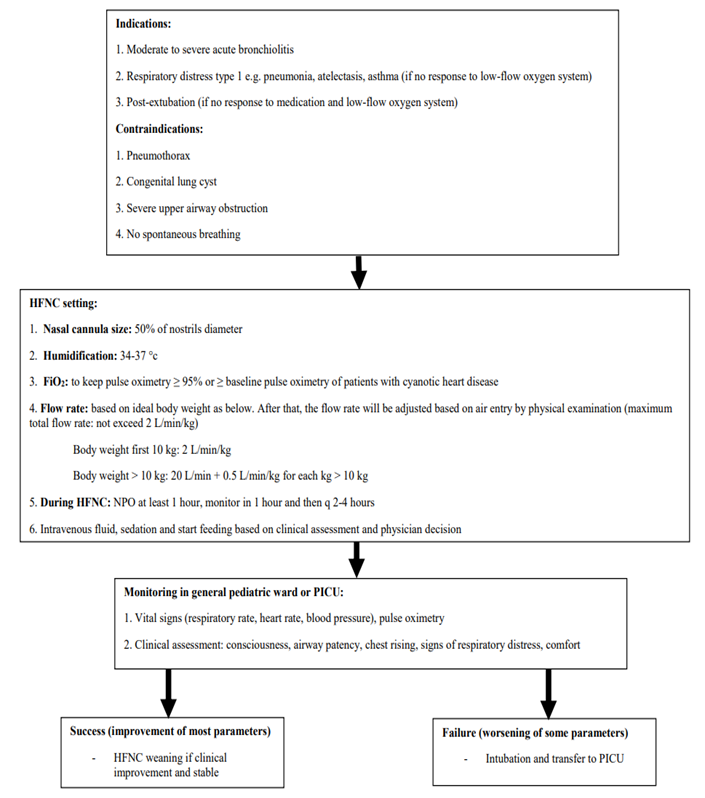

Supplement: Supplementary file 1 — Figure S1: HFNC initiation and monitoring in Pediatric Department, Chiang Mai University hospital [file CRJ-16-732-s001.tif]
